# Effect of preoperative oral rehydration before cesarean section on ultrasound assessment of gastric volume and intraoperative hemodynamic changes: a randomized controlled trial

**DOI:** 10.1186/s12871-023-02250-6

**Published:** 2023-08-30

**Authors:** Eriko Ijiri, Chie Mori, Tomoki Sasakawa

**Affiliations:** 1 Department of Anesthesiology and Critical Care Medicine, Asahikawa Medical University, Asahikawa, Japan; 2 Department of Anesthesiology, Tokyo Women’s Medical University, 8-1, Kawada-cho, Shinjyuku-ku, Tokyo, 162-8666 Japan

**Keywords:** Cesarean section, Gastric volume, Oral rehydration therapy, Preoperative fasting, Preoperative management

## Abstract

**Background:**

Cesarean section often requires an urgent transfusion load due to decreased blood pressure after spinal anesthesia. This prospective randomized study aimed to investigate whether a preoperative oral rehydration solution (ORS) stabilized perioperative circulatory dynamics.

**Methods:**

Sixty-three parturients scheduled for cesarean section under combined spinal epidural anesthesia (CSEA) were randomly allocated to one of three groups: Group O received 500 mL ORS before bedtime and 500 mL 2 h before CSEA; Group M received mineral water instead of ORS; and Group C had no fluid intake (controls). After entering the operating room, stomach size was measured using ultrasound. Blood samples were obtained, and CSEA was induced. Vasopressors were administered when systolic blood pressure was < 90 mmHg or decreased by > 20%. As a vasopressor, phenylephrine (0.1 mg) was administered at ≥ 60 beats/min heart rate or ephedrine (5 mg) at < 60 beats/min heart rate. The primary outcome was the total number of vasopressor boluses administered. Secondary outcomes were the cross-sectional area of the stomach antrum, maternal plasma glucose levels, serum sodium levels, total intravenous fluid, bleeding volume, urine volume, operative time, and cord blood gas values after delivery.

**Results:**

The total number of vasopressor boluses was lower in Group O than in Group C (*P* < 0.05). Group O had lower total dose of phenylephrine than Group C (*P* < 0.05). There were no significant differences between Group M and other groups. No differences were detected regarding secondary outcomes.

**Conclusions:**

In women scheduled for cesarean section, preoperative ORS stabilized perioperative circulatory dynamics. Neither ORS nor mineral water consumption increased the stomach content volume.

**Trial Registration:**

This trial is registered in the University Hospital Medical Information Network Clinical Trials Registry (UMIN000019825: Date of registration 17/11/2015).

## Background

Regional anesthesia (spinal, epidural, and combined spinal epidural [CSEA]) is the recommended standard practice for elective cesarean section because it offers a rapid onset and reliable surgical condition. In addition, it avoids the most common risks associated with general anesthesia, such as difficulty with airway management, pulmonary aspiration of gastric contents, and the negative effects of general anesthetics on the fetus. However, maternal arterial hypotension during regional anesthesia for cesarean section remains the main disadvantage, particularly for spinal anesthesia, and can result in fetal distress and maternal discomfort [1, 2]. The pathophysiology of hypotension following spinal anesthesia is believed to be caused by sympathetic vasomotor blockade, which causes arterial and arteriolar vasodilation and decreases systemic vascular resistance, resulting in hypotension. Venodilation also occurs, resulting in decreased cardiac preload, reduced cardiac output, and maternal hypotension. In addition, preoperative dehydration is a risk factor for hypotension during spinal anesthesia [3]. To attenuate spinal hypotension, many approaches have been investigated, [4–6] notably fluid loading, vasopressors, or a combination of both. Preoperative oral rehydration therapy (PORT) has been reported to prevent hemodynamic changes during anesthesia [7, 8].

Human and animal studies have found that carbohydrate loading before surgery leads to an improved response to surgical stress and postoperative conditions compared with traditional fasting guidelines. From such positive findings, PORT before elective surgeries has been recommended as an essential element of the enhanced recovery after surgery protocol. However, in general, late pregnancy causes delayed gastric emptying. The Practice Guidelines for Obstetric Anesthesia approved by the American Society of Anesthesiologists (ASA) suggests that women with uncomplicated pregnancies undergoing elective cesarean delivery should adhere to the same presurgical fasting guidelines as non-pregnant women, such as 6–8 h of no solid food and 2 h of no liquids before the scheduled surgical procedure [9, 10]; therefore, the safety of using oral rehydration solution (ORS) in women undergoing cesarean section remains unclear.

Our primary hypothesis with this prospective, randomized, open-label (but assessors are blinded), blinded-endpoint controlled clinical trial was to determine if preoperative intake of ORS (OS-1®) stabilized perioperative circulatory dynamics during cesarean section. We also measured stomach size using ultrasound to investigate the safety of ORS.

## Methods

### Ethics approval and consent to participate

This study was approved by the Ethics Committee of Kushiro Red Cross Hospital (Hokkaido, Japan) and is registered in the University Hospital Medical Information Network Clinical Trials Registry (UMIN000019825). The trial was conducted from February 2014 to July 2017 at Kushiro Red Cross Hospital, Japan, which is affiliated with the Department of Anesthesiology and Critical Care Medicine of Asahikawa Medical University. Written informed consent was obtained from all participants at least 24 h before the operation.

### Study participants

Parturients with no complications who were scheduled for elective cesarean section were recruited for this study. The inclusion criteria were healthy parturients aged > 18 years and with ASA physical status class II with term singleton pregnancies undergoing elective cesarean section under CSEA. All parturients were scheduled to enter the operating room at 9:00 am.

The exclusion criteria were emergency cesarean section, being scheduled for cesarean section under general anesthesia, multiple fetuses, abnormal pregnancy (such as placenta previa and placenta accrete), pregnancy complications (such as pregnancy-induced hypertension, gestational diabetes, bleeding disorders, and coagulopathy), fetal anomalies, and inability to undergo epidural anesthesia. In addition, failure to assess gastric measurement resulted in the exclusion of the participants from the data analysis.

### Randomization and masking

Eligible parturients were randomized into one of three groups using a web-based tool (http://www.graphpad.com/quickcalcs/index.cfm): ORS (Group O), mineral water (Group M), or fasted controls (Group C). Group O received 500 mL of ORS (2.5% carbohydrates, 10 kcal/100 mL; OS-1® Otsuka Pharmaceutical Factory, Japan) before bedtime the day before surgery and 500 mL 2 h before anesthesia induction. Group M received an equal volume of mineral water at the stated times. Group C was instructed not to have oral fluid intake for > 8 h before surgery. All parturients were allowed to eat and drink freely before bedtime. After bedtime, oral eating and drinking was forbidden, except for 500 mL of the appropriate drink in Groups O and M.

OS-1® contains water, glucose, and electrolytes and is packaged in a 500-mL plastic bottle; its composition is presented in Table 1.

**Table 1:** OS-1® composition

Volume	1000 mL
Energy	100 kcal
Carbohydrates	2.5% (glucose 1.8%; 100 mmol/L)
Sodium (Na^+^)	1150 mg (50 mmol/L)
Potassium (K^+^)	780 mg (20 mmol/L)
Magnesium (Mg^2+^)	24 mg (2 mmol/L)
Chloride (Cl^−^)	1770 mg (50 mmol/L)
Phosphorus (P)	62 mg (2 mmol/L)
pH	3.9
Osmolarity	Approx. 270 mOsm/L

Blinding of parturients was not feasible because of the taste of the drinks; instead, well-trained anesthesiologists were made unaware of patient allocation, and other study investigators analyzed the data.

### Gastric emptying assessment before anesthesia induction

On arrival to the operating room, the parturients were placed in the right lateral decubitus position. Before anesthesia induction, the gastric antral cross-sectional area (CSA) was measured using a Venue 40 (GE Healthcare, Tokyo, Japan) ultrasound system with a 2 to 5 MHz curvilinear array low-frequency 4 C-RS probe to measure gastric emptying [11–15].

**Fig. 1:**
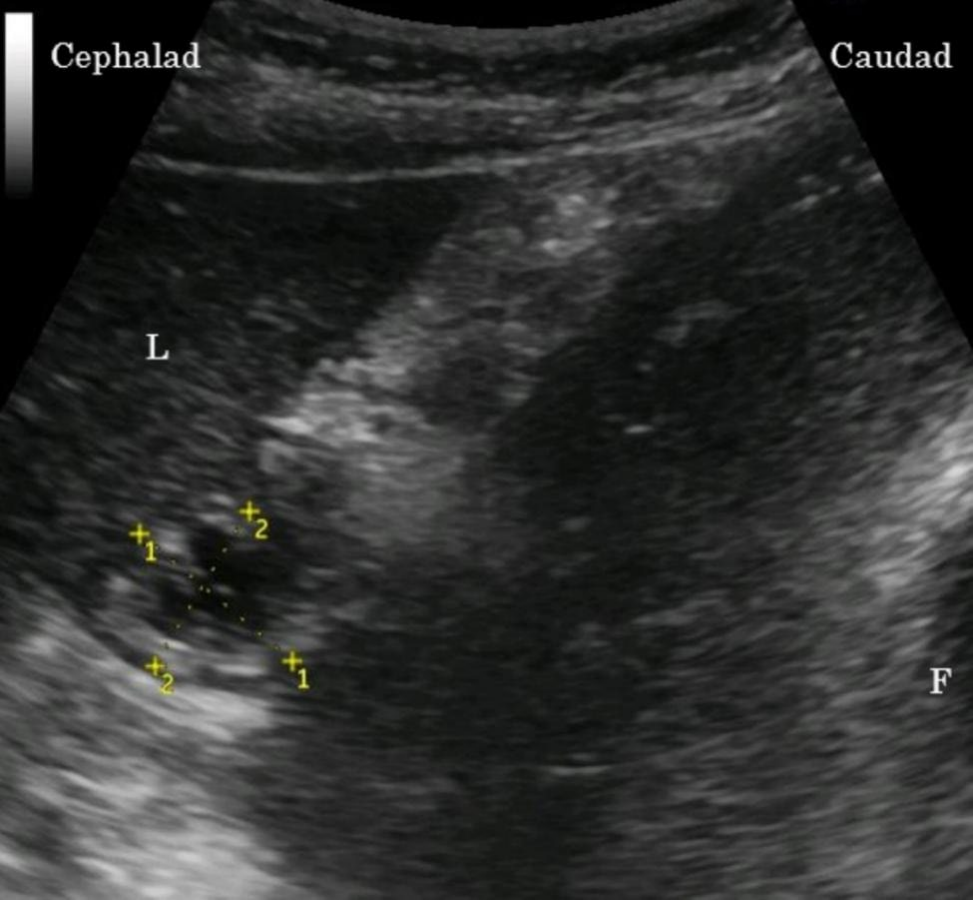
Ultrasonographic image of the gastric antrum The figure demonstrates two perpendicular diameters, line 1 and line 2, for cross-sectional area of the stomach antrum Line 1: Craniocaudal antral diameter Line 2: Anteroposterior antral diameter L, liver; F, fetal head

The gastric antrum was generally imaged in the parasagittal plane just right of the midline of the epigastric area in parturients, surrounded by the left lobe of the liver anteriorly and pancreas posteriorly. The CSA of the antrum was calculated according to the formula described by Bolondi et al. [16] using two maximum perpendicular diameters representing the surface area of an ellipse, as follows: CSA = AP × CC × π/4 (AP, anteroposterior diameter; CC, craniocaudal diameter) (Fig. 1).

### Anesthesia methods

After the ultrasound examination, routine monitoring, including heart rate (HR), blood pressure, electrocardiogram, and peripheral oxygen saturation, was implemented, and baseline values were recorded. Blood samples were collected after inserting a peripheral venous catheter, and a rapid infusion of 6% hydroxyethyl starch (HES) 130/0.4 (Voluven®, Fresenius Kabi Japan, Tokyo, Japan) was started; a total of 1000 mL was administered during surgery. Subsequently, we placed the parturients in the lateral decubitus position and attempted CSEA. All parturients underwent epidural catheterization before spinal anesthesia. An 18-gauge Tuohy needle was introduced using loss of resistance to air to confirm the epidural space. A 19-gauge epidural catheter was inserted through the epidural needle, 3–4 cm into the Th12/L1 epidural space. Instead of an epidural test dose, we injected 2 mL saline through the epidural catheter to prevent obstruction of the catheter by a blood clot. During skin closure, the parturient received continuous epidural infusion of 0.2% ropivacaine for postoperative analgesia.

All parturients received spinal anesthesia at the L3–4 interspace using a 25-gauge Quincke spinal needle (TOP Corp, Tokyo, Japan). Next, 0.5% hyperbaric bupivacaine (8 mg) mixed with fentanyl (20 µg) was administered intrathecally after free flow of cerebrospinal fluid was observed. According to the results of the pinprick tests, the surgeon commenced the operation once a sensory blockade above the T4 level was achieved. Vasopressors were administered when the systolic blood pressure was < 90 mmHg or decreased by > 20% of the baseline value. As a vasopressor, 1 mL phenylephrine (1 mg diluted to 10 mL [0.1 mg/mL] with normal saline) was administered for hypotension with HR ≥ 60 beats/min or 1 mL ephedrine (40 mg diluted to 8 mL [5 mg/mL] with normal saline) for hypotension with HR < 60 beats/min. We counted the administration of 0.1 mg of phenylephrine or 5 mg ephedrine as one dose.

The primary outcome of this study was the total number of vasopressor boluses and dose of vasopressors among the three groups. The secondary outcomes were the CSA of the stomach antrum, maternal plasma glucose levels, serum sodium levels, total intravenous fluid, bleeding volume, urine volume, operative time, and cord blood gas values after delivery.

### Data processing and analysis

A power analysis showed that 16 parturients per group would provide an α value of 0.05 and a β value of 0.1, based on the total dose of vasopressors during cesarean section in a pilot study of 10 parturients. The Kruskal–Wallis test was used for comparison between groups, followed by Dunn’s post hoc test for pairwise comparisons. Data are presented as median and interquartile range. All statistical analyses were performed using GraphPad Prism® version 6.01 (GraphPad Software, Inc., La Jolla, CA), and *P* < 0.05 was considered to indicate a statistically significant difference.

## Results

**Fig. 2:**
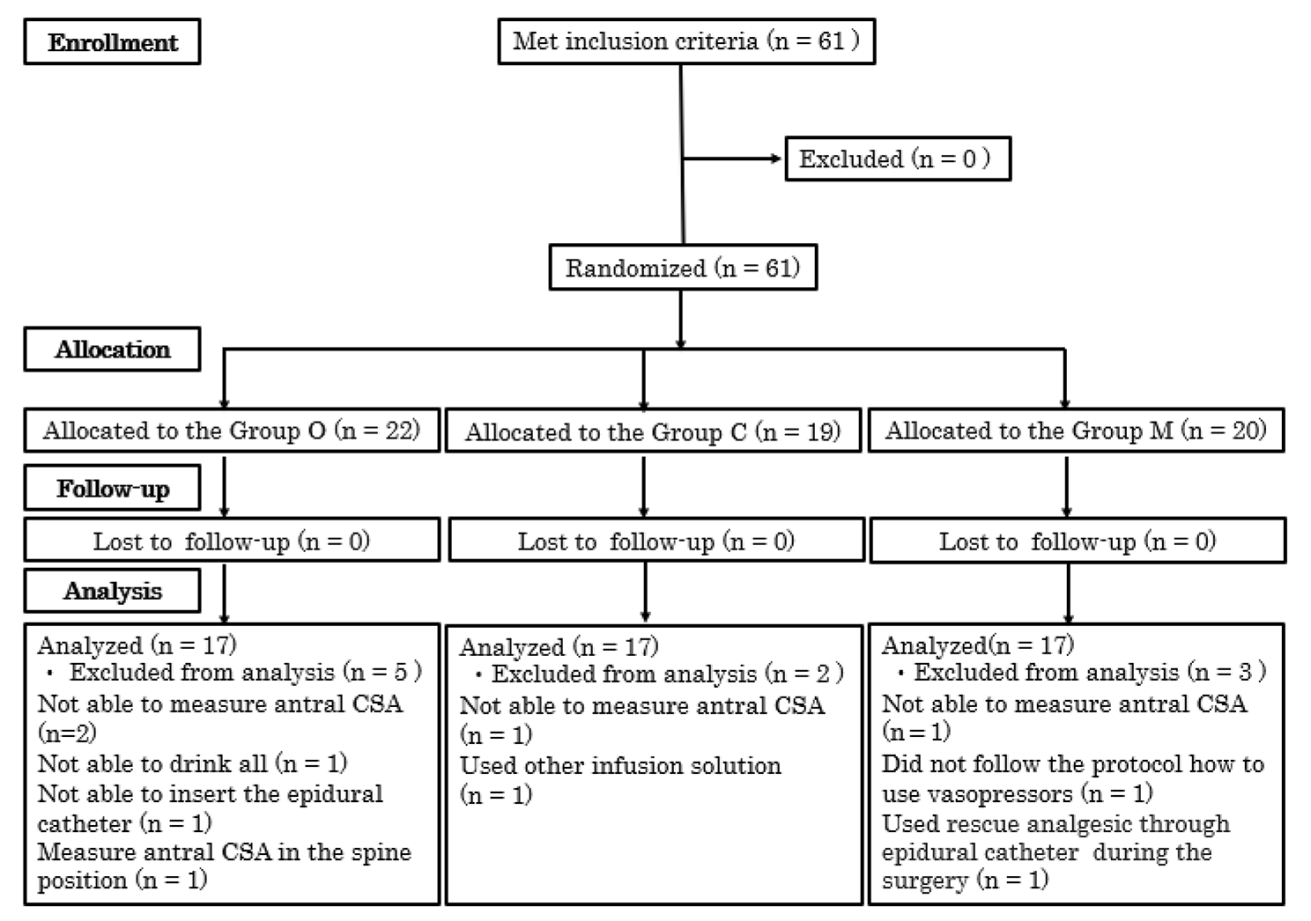
CONSORT flow diagram of study recruitment CSA, cross-sectional area

Sixty-one parturients were enrolled in this study and randomized into one of the three groups. The CONSORT diagram is shown in Fig. 2. We excluded five parturients in Group O, two parturients in Group C, and three parturients in Group M, as shown in the diagram. Finally, 17 parturients from each group were analyzed. The baseline characteristics of our study population and intraoperative data were compared between groups (Table 2).

**Table 2:** Baseline characteristics of study parturients

	Group O		Group C		Group M		*P*
	**(n = 17)**		**(n = 17)**		**(n = 17)**		
Age (years)	32.0	(27.5, 34.5)	34.0	(29.0, 37.5)	29.0	(27.0, 34.0)	0.203
Height (cm)	154.5	(151.5, 157.8)	157.0	(154.0, 159.7)	157.3	(153.5, 159.5)	0.162
Weight (kg)	58.8	(53.8, 65.0)	64.8	(52.3, 69.2)	65.4	(60.2, 69.4)	0.054
BMI (kg/m^2^)	24.7	(23.3, 26.2)	24.1	(21.6, 27.7)	26.1	(25.5, 29.3)	0.065

Values are presented as median [interquartile range]. Group O denotes the ORS group, Group C denotes the control group, Group M denotes the mineral water group, and n denotes sample size

### BMI, body mass index

**Fig. 3:**
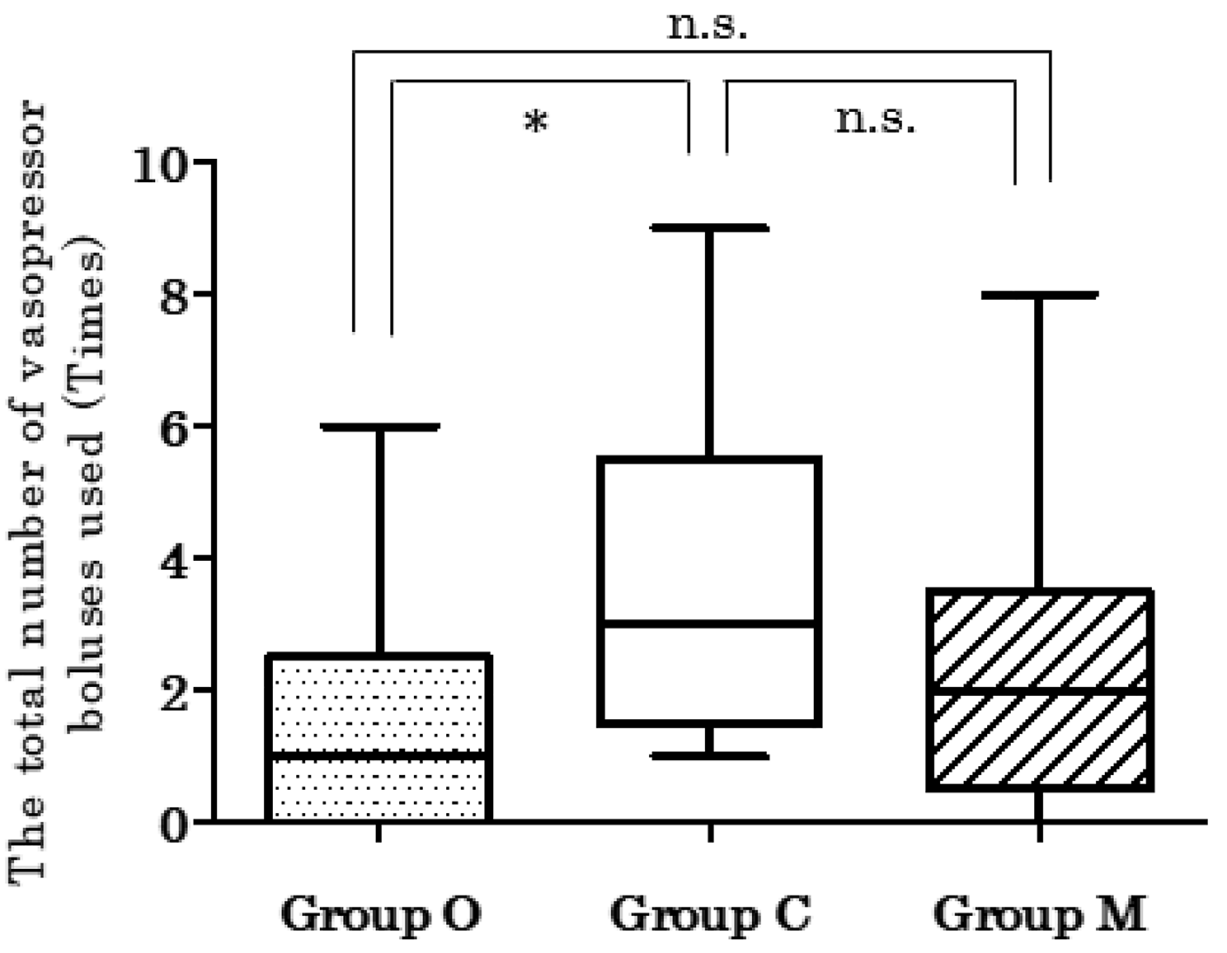
The total number of vasopressor boluses after inducing combined spinal epidural anesthesia. The probability was calculated using the Kruskal–Wallis test by ranks. A pairwise comparison was performed using Dunn’s test if the Kruskal–Wallis test was significant *Group O < Group C n.s., not significant

**Fig. 4:**
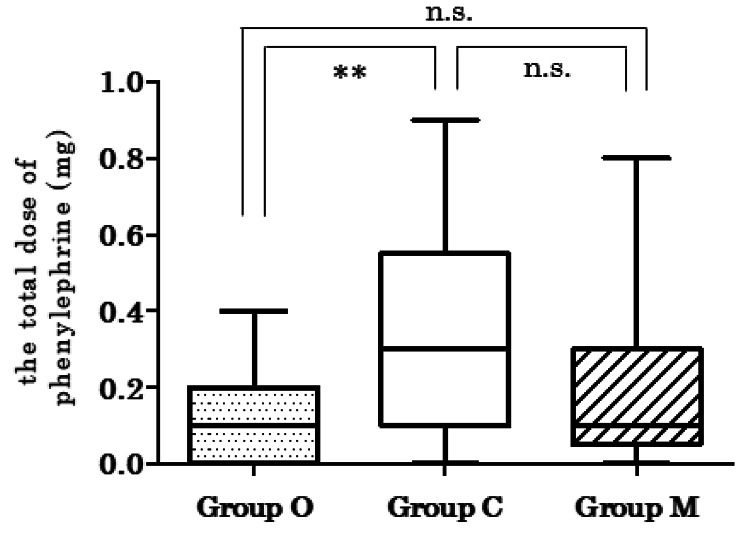
The total dose of phenylephrine after inducing combined spinal epidural anesthesia. The probability was calculated using the Kruskal–Wallis test by ranks. A pairwise comparison was performed using Dunn’s test if the Kruskal–Wallis test was significant **Group O < Group C n.s., not significant

The Kruskal–Wallis test indicated a statistically significant difference in the total number of vasopressor boluses used (*P* = 0.009). Post hoc comparisons using Dunn’s test showed that Group O required significantly fewer vasopressor boluses than Group C. There were no significant differences between Groups O and M or between Groups C and M (Fig. 3; Table 3). The difference in the total ephedrine dose was not significant between the groups. However, there was a statistically significant difference in the total phenylephrine dose (*P* = 0.017). Post hoc comparisons using Dunn’s test showed significant differences between Group O and Group C, with a lower dose used in Group O, whereas the other groups showed no significant differences (Fig. 4).

There was no significant difference in secondary outcome among the three groups (Table 3).

Furthermore, no side effects or serious complications were observed in any of the groups.

**Table 3:** Primary and secondary outcomes

			Group O		Group C		Group M		P-value
			(n = 17)		(n = 17)		(n = 17)		
**primary outcomes**		**The total number of vasopressor boluses(time)**	1.0	[0.0, 2.5]	3.0	[1.5, 5.5]	2.0	[0.5, 3.5]	**0.009**
		**The total dose of phenylephrine(mg)**	0.10	[0.00, 0.20]	0.30	[0.10, 0.55]	0.10	[0.05, 0.30]	**0.017**
		The total dose of ephedrine(mg)	0.0	[0.0, 0.0]	0.0	[0.0, 0.0]	0.0	[0.0, 2.5]	0.637
**secondary ** **outcomes**		Antral CSA(cm^2^)	2.9	[1.6, 5.0]	3.0	[1.0, 6.1]	2.8	[1.7, 4.8]	0.978
	Maternal venous	Glucose(mg/dL)	73.0	[68.5, 76.5]	74.0	[69.5, 80.5]	73.0	[66.5, 77.0]	0.531
		Sodium(mEq/L)	134.0	[134.0, 135.5]	135.0	[133.5, 136.0]	134.0	[133.0, 135.0]	0.245
		Total intravenous fluid(mL)	1300.0	[1150.0, 1425.0]	1200.0	[1100.0, 1425.0]	1250.0	[1125.0, 1600.0]	0.594
		Amount of bleeding(mL)	1020.0	[696.0, 1371.0]	823.0	[592.0, 1023.0]	923.0	[768.0, 1193.0]	0.327
		Urine Volume(mL)	200.0	[95.0, 350.0]	190.0	[100.0, 275.0]	160.0	[100.0, 325.0]	0.914
		Operative time(min)	58.0	[53.5, 70.5]	57.0	[51.5, 58.5]	56.0	[49.0, 66.0]	0.668
	Umbilical arterial	pH	7.3	[7.2, 7.3]	7.3	[7.2, 7.3]	7.3	[7.3, 7.3]	0.718
		PCO_2_(mmHg)	55.0	[46.0, 65.0]	57.0	[51.0, 63.5]	53.3	[50.6, 58.0]	0.659
		PO_2_(mmHg)	20.0	[14.0, 23.5]	17.2	[16.6, 21.2]	20.0	[15.9, 24.0]	0.801
		BE(mmol/L)	-2.0	[-2.0, 0.0]	-1.0	[-3.7, 1.0]	-1.9	[-3.7, 0.9]	0.923

Data are expressed as median [interquartile range]

CSA: cross-sectional area; BE: base excess; PCO_2_: partial pressure of carbon dioxide; PO_2_: partial pressure of oxygen

## Discussion

In this prospective randomized study of 51 parturients undergoing scheduled cesarean section, we found that PORT with OS-1® reduced the number of vasopressor boluses and phenylephrine doses during surgery compared with the fasting group. In addition, gastric emptying in healthy parturients was not delayed after drinking 500 mL of clear fluid 2 h before cesarean section compared to that in the fasting group. Similarly, a previous study concluded that PORT prevents hypotension after spinal anesthesia induction [7]. Another study reported that PORT increased the circulating blood volume and maintained a high cardiac index during the induction of general anesthesia [8]. To the best of our knowledge, this is the first study to evaluate the effect of preoperative oral rehydration on the incidence of spinal anesthesia-induced hemodynamic changes during scheduled elective cesarean section.

In this study, we used OS-1®, which meets the oral rehydration therapy guidelines recommended by the World Health Organization and is a balanced mixture of water, glucose, and electrolytes [17, 18]. Its composition was based on the guidelines of the American Academy of Pediatrics [19]. There are reports that preoperative intake of ORS prevents dehydration, [20, 21] and administration of ORS is believed to restore the circulatory volume. Three possible explanations can be considered. First, because ORS contains sodium and glucose, the active absorption of sodium and glucose by sodium-coupled glucose transporter-1 promotes the absorption of water in the small intestine [22–24]. Second, a solution with 45–60 mmol/L sodium and 80–110 mmol/L glucose resulted in effective fluid absorption, [25] and the composition of OS-1® was similar to this ratio. Finally, hypotonic solutions may promote increased water and solute absorption in the jejunum, and the osmolality of OS-1® is hypotonic, 270 mOsm/L.

Generally, parturients are at an increased risk of anesthesia aspiration. An increase in the intragastric pressure due to the gravid uterus, a relaxed gastroesophageal sphincter due to the increased progesterone level, and delayed gastric emptying during pregnancy contribute to the risk. For this reason, although recent guidelines recommend the intake of clear liquids at least 2 h before elective surgery, [9, 10] traditional prolonged preoperative fasting remains common, especially among the parturients, and we set the duration of fasting time of fasted controls (group C). Several previous studies have demonstrated the usefulness of the gastric content volume assessment by ultrasonography in parturients, [26–30] which is accurate despite being a simple, noninvasive technique.

Regarding the safety of PORT, we found that as the ORS is highly absorbable and has a short stagnation time in the stomach, ultrasound assessment of the gastric antral CSA in parturients did not increase. There was no apparent or potential risk of aspiration, vomiting, or other drink-related complications before, during, or after surgery. Therefore, we confirmed, by ultrasound, that 500 mL of ORS 2 h before surgery could be acceptable in parturients undergoing cesarean section under spinal anesthesia.

Preoperative infusion is said to be equally effective for the prevention of dehydration during the induction of anesthesia [31]. However, there are reports that excessive infusions cause intestinal edema, prolong the recovery of intestinal function, [32] and increase postoperative complications [33]; therefore, oral rehydration is preferable to reduce the perioperative infusion volume, including preoperative infusions. Oral rehydration is also superior to transfusion for optimizing fluid balance, such as supplying electrolytes and maintaining urine output. Furthermore, 6% HES 130/0.4 was used as an intraoperative infusion in this study, as it has been shown to be effective in preventing hypotension following spinal anesthesia for cesarean Sects [34, 35]. Generally, colloid fluids have some side effects, especially on the hemostatic system. However, Voluven® is a new HES with fewer side effects because of its low molecular weight [36].

In the present study, the ORS group showed significant differences from the fasting group in regards to the suppression of circulatory changes; however, ORS and mineral water did not show significant differences. Regarding excretion from the stomach, water and ORS were excreted at rates similar to those in previous studies on healthy adults. Thus, ORS is well excreted from the stomach; in addition, previous research has shown that ORS can correct electrolytes and reduce insulin resistance [37, 38]. Considering all these factors, ORS may be more beneficial than water.

Nonetheless, our study has some potential limitations. First, as it was impossible to obtain informed consent for emergent cesarean section because of the study protocol; all participants had scheduled elective cesarean sections. We also did not include parturients with complications or ASA physical status class ≥ III. This may limit the generalizability of our conclusions to more severe cases. Accordingly, further studies are needed to confirm whether the results would differ in such cases. Second, it was not a blinded clinical trial because of the taste of the drink, which may have increased the bias. However, the data were analyzed by a study investigator who was not involved in providing anesthesia. Third, although preoperative drinking could cause perioperative nausea or vomiting, this effect was not examined in the present study and is an issue for future research. Finally, sonographic gastric examination is often difficult, especially because pregnancy increases the technical difficulty.

## Conclusions

Our study showed that in women scheduled for cesarean section, preoperative oral rehydration with OS-1® stabilized perioperative circulatory dynamics and neither ORS nor mineral water consumed preoperatively increased the stomach content volume.

## Data Availability

The datasets used and analyzed during the current study are available from the corresponding author on request.

## Abbreviations

AP: anteroposterior diameter
ASA: American Society of Anesthesiologists
CSA: cross-sectional area
CSEA: combined spinal epidural anesthesia
CC: craniocaudal diameter
ORS: oral rehydration solution
PORT: preoperative oral hydration therapy
